# Rev. Thomas Waugh Belcher

**Published:** 1910-04-02

**Authors:** 


The death was announced on Monday last of the Rev. Thomas Waugh Belcher, M.D., M.S., D.D., rector of Frampton Cotterell, Bristol. He was educated at Trinity College, Dublin, where he took his B.A. degree in 1851, and after studying in the medical schools of Paris and Vienna, graduated at Dublin both in medicine and surgery, to which in 1856 he added the Oxford *ad eundem* degrees of M.A. and M.B. He was for a time physician to the Cork Fever Hospital, and served as surgeon-major in the Royal Cork Artillery Militia. He was also censor, examiner, and chief librarian of the Royal College of Physicians, Dublin, of which he was senior Fellow. In 1869 he forsook medicine for the Church, and was ordained deacon by Archbishop Tait (priest, 1870), and became curate of Charlton, Dover. In 1886 he became rector of Frampton Cotterell.

